# Language abnormalities in schizophrenia: binding core symptoms through contemporary empirical evidence

**DOI:** 10.1038/s41537-022-00308-x

**Published:** 2022-11-12

**Authors:** Xiao Chang, Wei Zhao, Jujiao Kang, Shitong Xiang, Chao Xie, Hugo Corona-Hernández, Lena Palaniyappan, Jianfeng Feng

**Affiliations:** 1 Institute of Science and Technology for Brain-Inspired Intelligence, Fudan University, Shanghai, China; 2 Key Laboratory of Computational Neuroscience and Brain-Inspired Intelligence, Fudan University, Ministry of Education, Shanghai, China; 3 MOE Frontiers Center for Brain Science, Fudan University, Shanghai, China; 4 Zhangjiang Fudan International Innovation Center, Shanghai, China; 5 MOE-LCSM, School of Mathematics and Statistics, Hunan Normal University, Changsha, PR China; 6 Shanghai Center for Mathematical Sciences, Shanghai, China; 7 Department of Biomedical Sciences of Cells & Systems, University Medical Center Groningen, University of Groningen, Groningen, The Netherlands; 8 Douglas Mental Health University Institute, Department of Psychiatry, McGill University, Montreal, Quebec Canada; 9 Robarts Research Institute, University of Western Ontario, London, Ontario Canada; 10 Lawson Health Research Institute, London, Ontario Canada; 11 Department of Computer Science, University of Warwick, Coventry, UK

**Keywords:** Schizophrenia, Diseases of the nervous system

## Abstract

Both the ability to speak and to infer complex linguistic messages from sounds have been claimed as uniquely human phenomena. In schizophrenia, formal thought disorder (FTD) and auditory verbal hallucinations (AVHs) are manifestations respectively relating to concrete disruptions of those abilities. From an evolutionary perspective, Crow (1997) proposed that “schizophrenia is the price that Homo sapiens pays for the faculty of language”. Epidemiological and experimental evidence points to an overlap between FTD and AVHs, yet a thorough investigation examining their shared neural mechanism in schizophrenia is lacking. In this review, we synthesize observations from three key domains. First, neuroanatomical evidence indicates substantial shared abnormalities in language-processing regions between FTD and AVHs, even in the early phases of schizophrenia. Second, neurochemical studies point to a glutamate-related dysfunction in these language-processing brain regions, contributing to verbal production deficits. Third, genetic findings further show how genes that overlap between schizophrenia and language disorders influence neurodevelopment and neurotransmission. We argue that these observations converge into the possibility that a glutamatergic dysfunction in language-processing brain regions might be a shared neural basis of both FTD and AVHs. Investigations of language pathology in schizophrenia could facilitate the development of diagnostic tools and treatments, so we call for multilevel confirmatory analyses focused on modulations of the language network as a therapeutic goal in schizophrenia.

“It seemed not improbable that the cortical centres that are the last organized, which are the most highly evolved and voluntary, and which are supposed to be located on the left side of the brain, might suffer first in insanity”.

James Crichton-Browne (1879)^1^, *On The Weight of the Brain.*

## Introduction

Schizophrenia is a neuropsychiatric disorder involving several language disturbances^2^. Disrupted speech productions (e.g., derailment, tangentiality, and thought block), reduced verbal output (the negative symptom of alogia), and aberrant speech perceptions (e.g., hearing “voices” in the absence of acoustic-linguistic stimuli) are hallmark symptoms and diagnostic criteria for schizophrenia^3,4^. Delusions are also viewed as a disturbance in the referential use of language^5^. Besides, several pragmatic, semantic and syntactic processing deficits are seen across all stages of schizophrenia (for reviews, see^6,7^). While these phenomena appear ostensibly varied, they can be conceptually traced to a shared pathophysiological and neurocognitive substrate: the language system^7–10^.

The study of the language system in schizophrenia has a long tradition. By examining the relative weight of patients’ two cerebral hemispheres, Crichton-Browne suggested that, in the course of schizophrenia development, the left hemisphere may suffer first^1^, implying that cortical language-related brain regions might be affected. Kraepelin described one form of dementia praecox as “an unusually striking disorder of expression in speech, with relatively little impairment of the remaining psychic activities”^11^. Bleuler argued that “looseness of associations” in thought, speech and other psychological functions is a core feature of schizophrenia^12^. From an evolutionary perspective, Crow proposed that a “saltational genetic change” which occurred between 100 and 250 thousand years ago allowed the two cerebral hemispheres to develop somewhat independently, laying the foundation for language to evolve^13^. Crow further considered that, arising from this evolutionary event, schizophrenia could be seen as an epiphenomenon of a failure to establish hemispheric specialization for language^8,13,14^. Led by DeLisi and colleagues’ work on genetic susceptibility and language network^8^, and later by Kircher’s^15^ and Kuperberg’s^6^ studies on anatomy of FTD and psycholinguistics respectively, the interest on language as a core domain in psychosis continued over time.

More recent studies have supported the possibility that an evolutionary modification of the human brain lies at the core of the occurrence of psychotic disorders. Xu and colleagues found that schizophrenia-associated genetic loci are more likely to be found in human accelerated regions (HAR), i.e., genomic regions that are “*highly conserved among nonhuman species but experienced accelerated substitutions in the human genome*”^16^. This aligns with Erady and colleagues’ findings, who reported that certain genomic features (novel open reading frames proximal to HARs) contributing to schizophrenia arise, in part, from the divergence between humans and other primates^17^. Furthermore, studies have reported that unique human brain connectivity differentiating us from chimpanzee significantly overlaps with brain structural dysconnectivity in schizophrenia^18,19^.

Broadly, evidence indicating that language dysfunctions constitute part of the pathophysiological and neurocognitive substrate of schizophrenia comes from at least three different types of studies. First, *neuroimaging studies* have shown that patients with FTD and AVHs were characterized by structural and functional abnormalities in language-related brain regions^20–29^. Likewise, *neuromodulation studies* (e.g., repetitive transcranial magnetic stimulation, rTMS) targeting extended language regions have been shown to alleviate auditory hallucinations^30–33^, as well as to ameliorate gesture performance in schizophrenia^34^. Second, *neurochemical studies* have provided clues to link language-related disturbances with known deficits in dopaminergic and glutamatergic systems in schizophrenia^35,36^. Third, *genetic studies* have implicated the enrichment of language-related genes in schizophrenia^37^, which might contribute to brain dysconnectivity, especially in the early stages of the illness^38^. Also, a developmental speech-disorder related to the gene FOXP2 has been associated with cognitive dysfunction^39^, psychotic speech profiles^40^, and reduced grey matter density^41^ in patients with schizophrenia, albeit not consistently^42^.

Notwithstanding these accumulating indicators of a disruption in the neural substrates of language in schizophrenia, a comprehensive synthesis is still lacking across these various levels of evidence. To bring together FTD and AVHs (i.e., two major domains of psychosis) as concurrently language-system symptoms in schizophrenia, in this study we examined neuroimaging evidence and neurotransmitter-level observations that have been reported in the language brain regions in schizophrenia. Moreover, we considered candidate genes that have been involved in both schizophrenia and language functions. We argue that a dysfunction of the language system is a critical feature of schizophrenia, and that we could obtain better diagnostic and treatment options to alleviate these symptoms in patients by investigating FTD and AVHs’ underlying mechanisms across several levels. Note that, throughout the article, the perisylvian network (i.e., the left inferior frontal gyrus [BA44, BA45]) and (posterior) superior temporal regions (BA42, BA22) are referred to as language processing regions as a whole. Nonetheless, we acknowledge that language processing further involves brain regions such as the premotor cortex, the frontal operculum, the middle temporal gyrus, and the angular gyrus^43–46^.

## Symptoms related to language and brain abnormalities

### Formal thought disorder (FTD)

FTD comprises clusters of atypical language productions which are interpreted as reflecting alterations in the neurocognitive processes that structure thoughts. Loosening of associations, derailment, tangentiality and incoherence are all included under the umbrella term of “positive forms of FTD”, while poverty of speech and slowed thinking are commonly called “negative forms of FTD”. In this dichotomous categorization, the “positive form” is considered to reflect a disorganization of the cognitive processes underlying FTD, whereas the “negative form” would reflect a reduction of such processes. Both forms have been related to poor long-term clinical outcomes^47,48^.

The prevalence of FTD in schizophrenia varies across studies and across disease stages, ranging from 27% to 80%^15^, but in two large-scale studies, FTD was observed in more than half of patients with chronic schizophrenia (50.39% and 72.7%, respectively)^49,50^. FTD may also affect individuals without schizophrenia, such as those at high-risk for psychosis, patients’ non-psychotic relatives, and patients with affective disorders^51–53^, suggesting that FTD is a transdiagnostic feature that is not specific to schizophrenia.

Neuropsychological studies have linked FTD to alterations in executive functions^54–58^. McGrath et al.^56^ proposed that deficits in initiation and planning of speech may contribute to poverty of speech, while a failure at maintaining information and inhibiting distractions could lead to positive FTD symptoms such as looseness of associations and derailment. Xu et al. demonstrated that poor sustained attention and planning in first-episode patients predicted residual FTD symptom severity at one-year follow-up^57^. Inhibitory control has been found to be impaired in patients with FTD compared to non-FTD patients^59^. In a recent review, it has been proposed that an excitatory/inhibitory imbalance at the microscale level could result in linguistic disorganization and impoverishment in schizophrenia^7^.

Other studies have investigated semantic processing dysfunctions in patients with FTD^60–65^. Doughty and Done systematically reviewed semantic impairments in schizophrenia^61^. The authors found a large effect for naming and verbal fluency tests (both phonemic fluency and category fluency), suggesting an impairment in semantic knowledge, semantic memory, and executive function in patients. Interestingly, some studies using semantic priming tasks have reported that patients with FTD showed a hyper-priming effect, despite general deficits in processing speed and various cognitive abilities^63–65^. In this type of studies, subjects are required to decide whether a target is a word or not (i.e., implementing a lexical decision task) while a semantic priming effect is elicited in some trials. This semantic priming effect refers to the facilitation of processing a target stimulus (e.g., the word “boat”) by providing a meaningful stimulus that precedes the target and shares features of meaning with the target (e.g., the word “ship”). The priming effect can then be calculated as the difference in reaction time between trials with related prime-target pairs and trials with unrelated pairs. The hyper-priming hypothesis would suggest that positive forms of FTD symptoms such as derailment and tangentiality can be understood as a failure to inhibit loosely associated concepts or word meanings that are stored in an individual’s semantic network. A meta-analysis on semantic processing has shown that increased semantic priming is only observed in patients with thought disorder, but not in patients with schizophrenia as a whole group^65^. Moreover, it has been suggested that the hyper-priming effect is restricted to indirectly related prime-target word pairs^66^. Yet, the specific level of hierarchical linguistic processing wherein the abnormal priming effect occurs in schizophrenia is still unclear^67^. Recently, a higher degree of semantic similarity that may result from the hyper-priming effect has been related to a model of reduced synaptic gain in Broca’s area and temporal semantic hub^62,68^, suggesting that a glutamate-centered excitation/inhibition imbalance plays a role in the lexical-semantic deficits seen in schizophrenia.

FTD has also been related to impaired pragmatic abilities^69–71^. Broadly, pragmatics concerns the actual use of language in socio-cultural constrained contexts of communication, focusing on the interpretation of literal, figurative and implicated meanings^72^. Patients with schizophrenia show impairments in understanding proverbs, metaphors, and irony, as well as in inferring communicative intentions^73,74^. Kuperberg et al. found that, compared to non-FTD patients and controls, patients with schizophrenia and FTD are less sensitive to pragmatic violations^70^. The severity of FTD symptoms has also been associated with pragmatic task performances^69,71^, although pragmatic deficits in schizophrenia may also relate to Theory of Mind impairments^75^.

### Auditory verbal hallucinations (AVHs)

AVHs can be loosely defined as speech-like perceptions of voices in the absence of external sources. Like FTD, AVHs constitute a transdiagnostic psychotic symptom, occurring in patients with affective disorders^76^, Alzheimer’s disease^77^, substance abuse^78^, and even in the healthy population^79^. The cross-sectional prevalence of AVHs in schizophrenia attains a range between 40% and 80% of chronic patients^80,81^, similar to what is reported for FTD. Studies have described that FTD and AVHs tend to co-occur in both patients with schizophrenia and non-psychotic subjects, suggesting that FTD and AVHs may share common neural abnormalities^82,83^.

Several neurocognitive models have been proposed to account for AVHs. To illustrate, here we mention only two. First, the source monitoring model suggests that AVHs occur when people experience self-generated inner speech as if it was externally generated^84,85^. In this account, the source monitoring abnormality would reflect disruptions in the corollary discharge^86^, which is a general mechanism to attenuate sensations from self-generated actions, thus distinguishing them from externally originated sensations^87,88^. Alternatively, the memory intrusion hypothesis proposes unwanted memories as the source of the hallucinations’ content^89,90^. In line with this, patients with auditory hallucinations exhibit deficits in inhibition of memories, and to have difficulties contextualizing them^89^. This is of relevance to the content of AVH often relating to traumatic experiences^91^.

## Neuroimaging evidence of impairments in the language circuits

Structural and functional abnormalities in language-processing regions are frequently found in neuroimaging studies of schizophrenia^38,92,93^. In schizophrenia, frontal and temporal lobe brain regions are the most affected areas, along with widespread cortical thinning and surface area reduction^94^. Cortical thinning in the language regions correlates with the severity of patients’ positive symptoms^94,95^. Previous brain-wide association studies (BWAS)^96^ identified that, in the early stages of schizophrenia, a functional dysconnectivity takes place in the inferior frontal gyrus^92,93,97^.

**Fig. 1:**
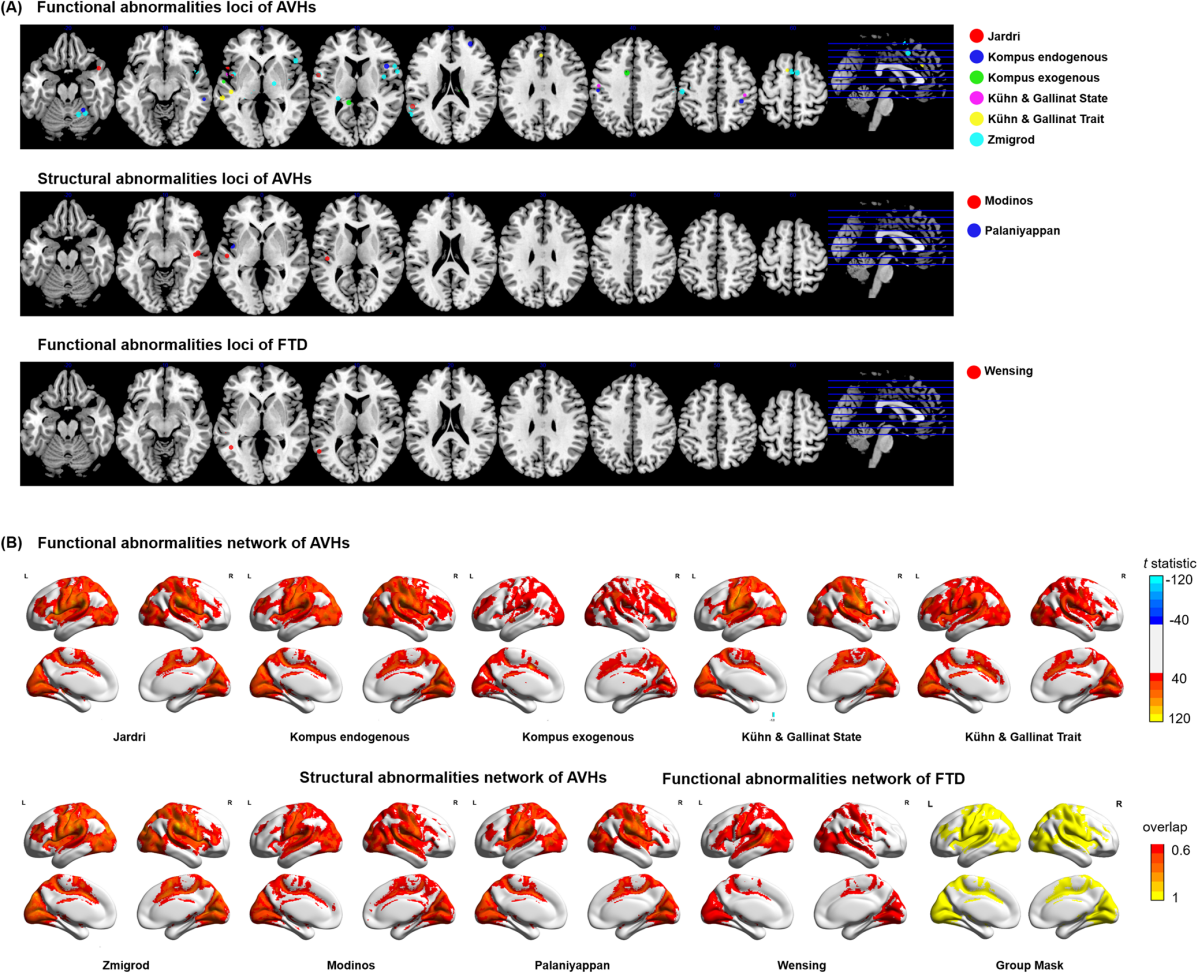
Brain abnormalities associated with formal thought disorders (FTD) and auditory verbal hallucinations (AVHs). A Significant loci retrieved from previous meta-analyses of FTD^23^ and AVHs^24,25,27–29,109^. A four-millimeter sphere is created centering on the reported loci. Colors indicate different meta-analyses. **B** T-statistic maps of brain regions connected to the activation loci of each meta-analysis (T threshold = 40 for illustration). A group mask was created based on binarized T-statistic maps above 60% of studies.

To provide a descriptive summary of brain abnormalities associated with FTD and AVHs, we searched PubMed for meta-analyses of neuroimaging studies on brain abnormalities in relation to FTD and AVHs (as of May 2022). Included meta-analyses are listed in Table 1. Reported significant loci from these meta-analyses are displayed in Fig. 1A, and listed in Supplementary Information (SI) Table 1.

**Table 1:** Meta-analyses of formal thought disorder and auditory verbal hallucinations^a^.

Author	Year	Imaging modality	Num. of studies	Num. of subjects	Meta-analysis methods	Significant threshold
*Formal thought disorder*						
Wensing et al.^23^	2017	task-based fMRI, PET	18 studies	165 SZ-FTD, 15 HC-FTD, 25 SZ-nonFTD, 132 HC	Activation likelihood estimation	cluster FWE *q* < 0.05 (cluster-forming threshold at voxel *p* < 0.001)
*Auditory verbal hallucinations*						
Modinos et al.^28^	2013	sMRI (grey matter volume)	9 studies	307 SZ-AVHs, 131 SZ-nonAVHs, 307 HC	Parametric Voxel-based Meta-analysis	cluster-level *q* < 0.001 uncorrected
Palaniyappan et al.^29^	2012	sMRI (grey matter volume)	7 studies	350 SZ	Signed Differential Mapping	*p* < 0.005, cluster extent 10 voxels
Jardri et al.^24^	2011	task-based fMRI, PET, SPECT	10 studies	68 SZ-AVHs	Activation likelihood estimation	FDR *q* < 0.05, cluster threshold = 200 mm^2^
Kühn and Gallinat^27^	2012	task-based fMRI, PET	State studies:10; Trait studies: 8	State: 85 SZ-AVHs; Trait: 81 SZ-AVHs, 39 SZ-nonAVHs, 69 HC	Activation likelihood estimation	FDR *q* < 0.01, cluster threshold = 100 mm^2^
Kompus et al.^25^	2011	task-based fMRI, PET	Endogenous studies: 12; Exogenous studies: 11	Endogenous: 103 SZ-AVHs; Exogenous: 204 SZ-AVHs, 170 HC	Activation likelihood estimation	FDR *q* < 0.05, cluster threshold = 200 mm^2^
Zmigrod et al.^109^	2016	fMRI, PET	13 studies	190 AVHs (SZ, Psychosis, HC, Other diagnoses)	Activation likelihood estimation	FDR *q* < 0.05, cluster threshold = 200 mm^2^

^a^Meta-analyses were retrieved from PubMed search: (“formal thought disorder” OR “auditory hallucinations” OR “auditory verbal hallucinations”) AND (neuroimaging OR (“brain imaging”) OR (“magnetic resonance imaging”) OR MRI OR (“positron emission tomography”) OR PET). The article type was limited to meta-analyses published in English between January 1991 and August 2021.

### Brain abnormalities in relation to FTD

Only one fMRI meta-analysis on FTD was found from the literature search^23^. The authors reported two significant loci showing abnormal activation: one in the left posterior middle temporal gyrus, and another in the left superior temporal gyrus (Fig. 1A and SI Table 1). Two systematic reviews implicated other brain functional abnormalities in FTD, including the bilateral inferior frontal gyri, anterior cingulate cortex, striatum and cerebellum^20,22^. Systematic reviews reported grey matter volume reduction in FTD, including bilateral superior temporal gyri, inferior frontal gyri, inferior parietal lobe, orbitofrontal cortex, cerebellum, nucleus accumbens and amygdala-hippocampal region^20,21^. Previous studies suggested that structural abnormalities in the language regions are associated with semantic task performances^98^ and the severity of FTD^99^.

The neuroimaging findings are consistent with the above-mentioned neuropsychological accounts of FTD, in that structural and functional abnormalities in the superior and middle temporal gyri constitute core deficits of FTD. Additional abnormalities in other brain regions subserving executive functions (anterior cingulate cortex and lateral prefrontal cortex) and motivation (orbitofrontal and medial prefrontal cortex) have also been related to positive FTD^100,101^, whereas insula, precuneus and frontocingulate abnormalities more closely relate to negative FTD symptoms^21,99,102^. Of notice, in the case of reduced connectivity between core language-processing regions, inefficient or maladaptive engagement of non-language regions may contribute to a more severe manifestation of FTD^103^.

A few diffusion-weighted imaging (DWI) studies have been conducted in schizophrenia patients with FTD. In line with the structural studies, fibers connecting the language regions, including the middle longitudinal fasciculus^104^, cingulum^105^ and uncinate fasciculus^106^, have been found to show reduced integrity, which is associated with FTD severity. Associations with other tracts, such as the corpus callosum^107^ and internal capsule^108^, have also been reported. Yet, in general, the results of DWI findings in FTD vary across studies^20,105^. Thus, at present, the sensitivity and specificity of white matter impairment in relation to FTD remains unclear.

### Brain abnormalities in relation to AVHs

Two sMRI meta-analyses of AVHs were retrieved from the literature search^28,29^. The most commonly reported structural abnormalities involve the bilateral superior temporal gyri, followed by the right middle temporal gyrus and the right insula (Fig. 1A and SI Table 1). For the functional studies, four fMRI meta-analyses of AVHs were retrieved from the existing literature^24,25,27,109^ (Fig. 1A and SI Table 1). They all showed abnormal activation converging on the bilateral superior temporal gyri, the middle temporal gyri and the inferior frontal gyrus. Other functional abnormalities in AVHs included the post-central gyri, the supramarginal gyri, the insula, the anterior cingulate cortex, the thalamus, the hippocampus, and the cerebellum^24,25,27,109^. Some studies reported an activation of speech-production and speech-perception regions, as well as their right hemisphere homologues^110–112^. Other studies have suggested that the altered activity of the speech-perception regions is modulated by the inferior frontal gyrus, the anterior cingulate cortex and the insula (the salience network). An altered connectivity among these brain regions may then underlie patients’ impairment in source-monitoring and salience-detection functions, resulting in the misattribution of internal voices as externally generated^110,112–114^. It is worth noting that functional abnormalities in the superior temporal gyrus in patients occur across different imaging conditions, including the hallucination periods^115^, during language tasks performance^25,27^, and in the absence of external tasks^116,117^, suggesting that this abnormality might have a primary role in the occurrence of AVHs.

DWI studies generally reported fractional anisotropy (FA) changes in the left arcuate fasciculus, which is the major association tract connecting the inferior frontal gyrus and the superior temporal gyrus. Parallelly, both increased^118,119^ and decreased FA^120–122^ were reported in patients with AVHs, compared to non-AVHs patients and healthy controls. As mentioned in the section Auditory verbal hallucinations (AVHs) earlier, this impairment in the arcuate fasciculus may give rise to AVHs through disrupted corollary discharge. Other studies also implicate the corpus callosum in AVHs, especially the section connecting the bilateral auditory cortex^120,123^.

### Brain abnormalities overlapping between FTD and AVHs

To quantify the degree of network-level overlap of brain abnormalities between FTD and AVHs, we conducted a recently proposed network mapping analysis approach^124–126^. This approach uses focal loci as seed regions to derive brain networks based on a normative human connectome (see Supplementary materials). To do this, first we retrieved focal loci from previous meta-analyses on FTD and AVHs^23–25,27–29,109^ (Table 1). Then, brain regions connected to the seed regions of each meta-analysis were determined by comparing the *z*-transformed connectivity with zeros using a one-sample *t*-test. T-statistic maps of each meta-analysis were set at a range of thresholds (T = 5–40, corresponding voxel-wise *p* = 1.7 × 10^−6^ ~ 1.8 × 10^−215^, *df* = 1095), and binarized to create a group mask (above 60% of studies) (Fig. 1B). This group mask represents the spatial distribution of a hypothetical connectome that has a high probability of being implicated in individuals with both AVHs and FTD (as in the case of schizophrenia). The results of this analysis revealed that functional networks of FTD and AVHs are indeed highly overlapping (Dice index ranging from 0.70 to 0.99, for T thresholds at 5–40, SI Table 2). When the overlap was restricted to a meta-analysis of functional imaging in AVHs^109^ and FTD^23^ only, we observed a 68.8% overlap. These results suggest that the neural substrate of FTD and AVHs overlaps considerably, affecting brain regions including the bilateral inferior frontal gyrus, the superior temporal gyrus, the pre- and post-central gyrus, the insula, the middle cingulate cortex and the occipital regions. Note that, since FTD and AVHs are simultaneously present in many patients, we could not determine if the shared neural abnormalities reported here partly relate to their concurrent presence in patients.

### Brain abnormalities in the course of illness development

If abnormalities in the language-processing frontal and temporal gyri are central to the pathology of schizophrenia, it becomes critical to understand the developmental course of such aberrations. Meta-analyses of longitudinal studies in schizophrenia have shown that patients exhibit an excessive grey matter volume loss in the frontal cortex and superior temporal gyrus^127,128^, which is related to symptoms severity and clinical outcomes^129,130^. These neural abnormalities occur even before the illness onset. In parallel, studies on high-risk individuals^131^, childhood-onset schizophrenia^132^, and first-episode schizophrenia patients^133,134^ have reported volume reductions in the frontal and temporal cortex. Consistently, other studies have found that patterns of brain functional abnormalities differ between the early stage and the chronic phase of the illness: first-episode patients show more functional dysconnectivity in the left inferior frontal gyrus, especially in males^38,92,93^, whereas patients with longer illness durations exhibit widespread dysconnectivity^96^. A study of medication-naive patients with varying illness durations also showed an accelerated cortical thinning in the prefrontal and temporal areas in patients^135^. Overall, these results indicate that, in the course of schizophrenia, frontal and temporal language-processing brain regions are affected since the early stages of the illness.

## Neurochemical and genetic basis of language abnormalities

### Neurochemical abnormalities

Over the decades, a dominant theory of schizophrenia has posited that dopaminergic dysfunction is at the core of the disorder^136–138^. Specifically, it has been shown that patients with schizophrenia exhibit a higher level of synthesis, release and binding of dopamine in subcortical regions, which can be treated with antipsychotics blocking dopamine D2 receptors^139,140^. Though antipsychotics alleviate the severity of AVHs and FTD, the majority of patients still show residual symptoms. This suggests that dysfunction in other neurotransmitter systems may play a role as well in their maintenance^141,142^.

The high dopaminergic level in subcortical regions has been reported to correlate with a dysregulation of glutamate in cortical areas^143^. Glutamate has been recently posited to play key roles in the physiology of both typical language processing and its disorders^144^. Moreover, previous studies have suggested that alterations in the glutamate system in frontal and temporal regions may underlie the occurrence of AVHs and FTD^35,36,145,146^.

It has been reported that, compared to patients without hallucinations, patients with auditory hallucinations have a higher level of glutamate/glutamine in the superior temporal gyrus and lateral prefrontal cortex^35^. Besides, patients’ hallucinatory symptoms correlated with a higher level of glutamate in the left superior temporal gyrus, and a lower level of glutamate in the anterior cingulate cortex, suggesting an imbalanced glutamate interaction between cortical regions in hallucinating patients^146^.

In terms of an association between glutamate and FTD, a pharmaco-fMRI study on healthy subjects showed that ketamine (glutamate NMDA receptor antagonist) elicited transient FTD symptoms, disrupting participants’ lexical and semantic verbal fluency^36^, and their free flowing speech^145^. Also, in these healthy participants, ketamine induced a higher correlation between speech production and brain activation in the right middle and inferior temporal gyri, similar to the brain activation in the superior temporal cortex observed in patients with FTD^147^. Interestingly, ketamine produces a cortical disinhibitory effect that disrupts the selective pyramidal neuronal tuning, which is required for working memory maintenance^148^. Moreover, ketamine, when administered to patients with prior experience of psychosis, results in the re-emergence of AVHs and FTD features in particular^149^. More direct examinations of the glutamate dynamics and FTD in patients are needed^150^, since pharmaco-fMRI studies in healthy participants only provide indirect evidence of the involvement of glutamate in the occurrence of FTD.

Overall, growing evidence points to the possibility that a glutamatergic disruption underlies the occurrence of AVHs and FTD in schizophrenia. Yet, disentangling the specific role that glutamate plays in typical language processing, on the one hand, and the occurrence and maintenance of AVHs and FTD, on the other, warrants further research.

### Genes related to schizophrenia and language

Schizophrenia’s heritability is estimated to be 79%^151^. Of notice, both FTD and AVHs are more prevalent in first-degree relatives of patients with schizophrenia than in first-degree relatives of healthy controls, suggesting that genetic variants contribute to the etiology of these symptoms^152,153^.

**Fig. 2:**
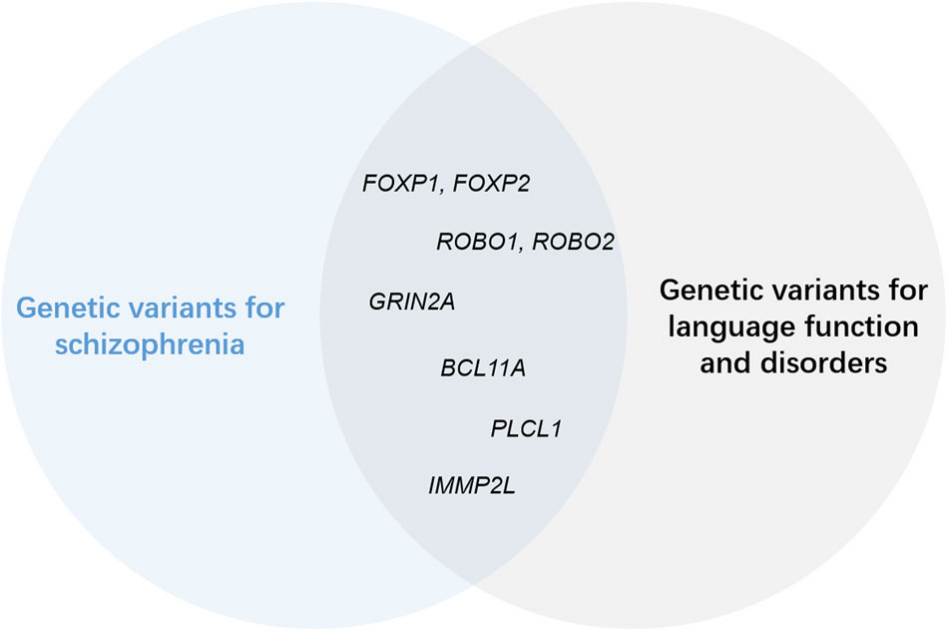
Shared genetic risk variants for schizophrenia and language disorders. Schizophrenia risk genes were retrieved GWAS catalog database (https://www.ebi.ac.uk/gwas/) using trait label “schizophrenia” (EFO ID: 0000692). Language-related genes were collated from two recent reviews^158,159^.

In conducting our review, we looked for studies examining schizophrenia-risk genetic variants that are parallelly implicated in both the development and dysfunction of language, and we also retrieved genetic studies specifically focused on FTD and AVHs in schizophrenia^154–157^. To systematically obtain the genetic variants that overlap between schizophrenia and language disorders, we first retrieved language-related genes from two recent reviews^158,159^. Schizophrenia-risk genes were subsequently identified from the genome-wide association studies (GWAS) catalog, including 1450 risk loci mapped genes. In total, we could identify 8 genes that overlap between and have been independently associated with both schizophrenia and language and its related disorders (Fig. 2). These include forkhead box P1, P2 (*FOXP1*, *FOXP2*), roundabout guidance receptor 1,2 (*ROBO1*, *ROBO2*), glutamate ionotropic receptor NMDA type subunit 2A (*GRIN2A*), BAF chromatin remodeling complex subunit (*BCL11A*), inactive phospholipase C-like 1 (*PLCL1*) and inner mitochondrial membrane peptidase subunit 2 (*IMMP2L*).

*FOXP1* and *FOXP2* encode transcriptional factors of the forkhead box family. Both genes have been associated with neural development and language evolution^160^. Risk alleles of *FOXP2* have been reported to contribute to auditory hallucinations in schizophrenia^161–163^. A recent study reported that a polygenic risk score obtained from *FOXP2* genetic clusters correlated with functional connectivity in the inferior frontal gyrus in first-episode schizophrenia patients with short illness duration^38^. Another study found that the *FOXP2* risk allele is associated with decreased gray matter volume in several brain regions, including language regions, in patients with schizophrenia^164^.

The *ROBO* gene family encodes proteins that are involved in axon guidance and cell migration, especially midline crossing and axons projections in the forebrain. The *ROBO2* gene has been related to the size of expressive vocabulary during language acquisition^165^, schizophrenia^166^, and handedness^167^. A different gene, *GRIN*, encodes subunits of NMDA receptors, with *GRIN2A* specifically encoding glutamate-binding GluN2 subunit^168^. The *GRIN2A* has been associated with several neurodevelopmental disorders, including speech disorders, seizures, autism and schizophrenia^169^. Now, *BCL11A*, also known as B-cell CLL/lymphoma 11 A, is a zinc finger transcription factor which mediates effects of the glutamate neurotransmitters on axonal branching and neurite outgrowth^170^. *BCL11A* influences early neurodevelopment and has been implicated in schizophrenia, intellectual developmental disorder, and expressive language development^171,172^. Aside, the *PLCL1* protein regulates efficacy of GABAergic neurotransmission^173^, and it has been associated with language development and risk for schizophrenia^174,175^. Finally, *IMMP2L* encodes the inner mitochondrial membrane peptidase subunit 2-like protein, which has been reported in schizophrenia, autism, and other neurodevelopmental disorders^176^. An animal study showed that *IMMP2L* knockdown mice exhibited sex-specific changes in locomotion activity and social interaction, which are symptoms of autism and Gilles de la Tourette syndrome^177^.

Overall, this accumulated evidence suggests that schizophrenia^178–181^ and language disorders^144^ share genetic variants that play a role in the early neural development and are likely associated with deficits in key neurotransmitter systems.

## Potential clinical applications of the analysis of speech

### Diagnostic and prognostic tools

In schizophrenia, features of distinctive language anomalies allow automatic linguistic analysis tools to discriminate between patients’ and healthy controls’ speech. Compared to control subjects, patients’ speech is characterized by longer pauses, reduced semantic coherence, lower sentence complexity and several anomalies at the phonetic, syntactic, semantic, and pragmatic levels^182^. Recent studies have shown that, implemented along with machine learning algorithms, automated speech analysis tools might help to reach a diagnosis of schizophrenia^183^ and to predict psychosis conversion among high-risk individuals^184^. A study focused on clinical high-risk individuals showed that a decrease in semantic coherence, greater variance in that coherence, and a reduction in usage of possessive pronouns were predictive features of psychosis conversion, reaching 79% accuracy in the test dataset^184^. Another study reported that patients with schizophrenia-spectrum disorders could be distinguished from healthy controls with 85% cross-validated accuracy using a set of measures related to connectedness across words^185^. Altogether, the existing evidence suggests that analyzing patients’ speech using automatic linguistic tools might help to reach clinical diagnoses and to predict conversion to psychosis, although a series of obstacles remain^186^.

### Treatments targeting language-processing brain regions

As FTD and AVHs have been shown to be associated with abnormalities in language-processing brain regions, non-invasive treatment strategies targeting these regions have been proposed. One commonly applied method is repetitive transcranial magnetic stimulation (rTMS), which generates a brief, high-intensity magnetic field that stimulates the brain tissue^187,188^. Studies have just started revealing the neural mechanism of rTMS treatment. Vercammen et al. found that 1-Hz rTMS targeting the left temporoparietal cortex enhanced functional connectivity strength between the target site and the right insula^189^. Arterial spin labeling (ASL) studies have shown that an improvement in clinical symptoms after rTMS treatment was accompanied by a reduction in blood flow in language brain regions^190^. Blood flow in the superior temporal gyrus further distinguished responders and non-responders to rTMS^191^. All these studies demonstrated that rTMS to language brain regions would modulate brain activity and connectivity between the target site and other brain regions.

While the rTMS treatment for FTD has been poorly studied^192^, many studies have investigated the efficacy of the rTMS to reduce AVHs, normally targeting the left temporoparietal cortex (including the superior temporal gyrus and the temporoparietal junction)^33^. While targeting the left temporoparietal cortex, some studies have reported a positive effect of the rTMS to reduce AVHs severity^32,33,193^, whereas others reports showed no superior benefit of real stimulation over sham conditions^194–196^. The inconsistency may reflect the heterogeneity of sampled patients (for example, in the degree of resistance to antipsychotics and illness duration), and stimulation protocols (e.g., duration and frequency of the stimulation). Two meta-analyses found that low-frequency (1 Hz) rTMS may achieve better efficacy, as this frequency can reduce hyperactivity of temporal areas in patients with AVHs^30,31^. Importantly, when rTMS has been applied on the dorsolateral prefrontal cortex (DLPFC), it did not improve language fluency^197^ nor AVHs^192^, which might be expected, mainly considering that the DLPFC seems to be involved primarily in pragmatic language processing^198^.

Broca’s area, a major player in the language circuit critical for verbal and non-verbal communication, has also been a site targeted by rTMS in patients with schizophrenia and AVHs, although results have been negative^194^. Nevertheless, co-speech gestures (i.e., non-verbal communication components) that are impaired in patients^199–201^ and in high-risk individuals for psychosis^202^ could be improved after Broca’s area stimulation^34^. However, more studies are still needed to explore different stimulation strategies and distributed target sites to fully take advantage of rTMS to alleviate AVHs and FTD.

## Future directions

**Fig. 3:**
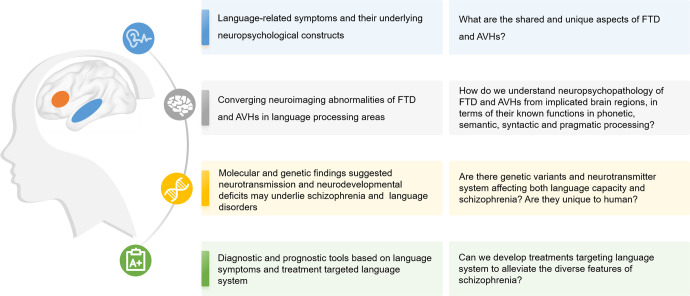
Multilevel evidence of language disturbances in schizophrenia. On the left, we list the focus of the current review ranging from behavioral symptoms, neuroanatomy, molecular-genetic mechanisms and potential clinical applications focused on language domain. On the right, from top to bottom are the outstanding questions pertinent to the overlap between formal thought disorder and auditory verbal hallucinations in schizophrenia, ranging from mechanistic aspects to treatments.

Different lines of research have gathered evidence of language disturbances in schizophrenia (Fig. 3 and Table 2). In this review, we mainly focused on the pathology of the language system in schizophrenia, yet other higher-order brain networks (e.g., the default mode network, the executive control network, and the salience network) have also been involved in FTD and AVHs^7^. Neurocognitive studies have reported that semantic and pragmatic abnormalities, as well as executive dysfunctions, are related to both FTD and AVHs^59,113^. Pragmatic deficits have also been related to Theory of Mind impairments^75,203^ (but see^204^). Overall, the current evidence suggests that both abnormalities in language-related regions and connectivity between language regions and other higher-order brain networks are impaired in schizophrenia. To determine how regional abnormalities and dysconnectivity across higher-order brain networks may give rise to FTD and AVHs, longitudinal and intervention studies are needed. For instance, studies could focus on how rTMS targeting higher-order brain networks might affect the language system over time in patients with schizophrenia and AVHs and/or FTD. Of notice, collecting speech samples in a harmonized manner will be important to conduct a deep phenotyping of language in such studies.

**Table 2:** Summary of findings across levels of analyses.

Levels of analyses	Formal thought disorder (FTD)	Auditory verbal hallucinations (AVHs)
Neurocognitive	• A transdiagnostic symptom with a positive and negative form; • FTD is related to executive functions such as inhibitory control, sustained attention and planning; • FTD is also related to semantic and pragmatic deficits	• A transdiagnostic symptom, tend to co-occur with FTD; • AVH is related to impaired monitoring function and likely failure to inhibit unwanted memories
Neuroanatomical	• Functional abnormalities involve inferior frontal gyrus, medial orbital frontal cortex and middle temporal gyri; • Structural abnormalities in left posterior superior temporal gyrus; • Structural connectivity studies are less consistent, implicating fibers connecting the language regions and also other fibers	• Abnormal activation during AVHs or auditory tasks in more widespread areas in bilateral superior and middle temporal gyri, inferior frontal gyri, post-central gyri, anterior cingulate cortex insula and cerebellum; • Structural abnormalities in the bilateral superior temporal gyri; • Structural connectivity studies mainly reported arcuate and uncinate fasciculus
Neurochemical	• Partially treated by antipsychotics • NMDA antagonisms elicits transient FTD symptoms and brain activation in the right middle and inferior temporal gyri	• Related to excessive dopamine in the striatum, • Partially treated by antipsychotics • Relates to higher level of glutamate/glutamine in the superior temporal gyrus and lateral prefrontal cortex
Genetic	• Genes implicated in both language function and schizophrenia mainly involve in early neural development and neurotransmission. Some genes are directly related to structure and function of language regions	

Neuroimaging studies have shown that both structural and functional abnormalities underlie patients’ AVHs^24–29^ and FTD^20–23^. FTD has also been frequently found in non-clinical voice-hearers (i.e., individuals with AVHs but no diagnosable psychiatric conditions)^82,83^. However, an elucidation of the shared and distinct neural basis of these syndromes is still missing. Longitudinal developmental brain imaging studies from both clinical and non-clinical samples with thorough phenotyping of AVHs and FTD will be critical to unpack the brain-level mechanisms of these syndromes. A valid and reliable characterization of the mechanisms underlying schizophrenia would have to account for how AVHs and FTD ‘come together’ in a single individual.

Genetic studies have found hundreds of genetic risk loci and structural variants for the phenotype of schizophrenia^205,206^ and for the faculty of language^207,208^. However, few studies have directly examined whether there is a shared genetic basis underlying these behavioral traits^37^. Moreover, more work is needed to further understand how this genetic basis is influenced by environmental exposure across the lifespan^209^, and how the genetic knowledge about schizophrenia might be key to offering more effective treatments against this disorder^210^.

The innate capacity of large-scale brain networks involved in higher-order functions such as language to adaptively reorganize in the face of dysfunction is becoming increasingly clear with modern neuroimaging and neuromodulation studies^211^. In the case of the language network, this is likely to occur through interhemispheric interactions^212^, as well as through contributions from domain-general regions. Such adaptive network plasticity not only supports recovery after damage, but also forms the robust basis for rehabilitative treatment approaches. Considering this, further seminal work is needed for us to develop and test what might be called a “Language Network Modulation” therapy targeting FTD and AVHs in schizophrenia. The success of such an approach may relate to the neuroimaging, neurochemical, linguistic, and genetic profile of the affected individual.

## Conclusion

Our review focused on current findings from different levels of analyses about language disturbances, FTD and AVHs among patients with schizophrenia. Neuropsychological studies indicate shared deficits in speech processing and its interaction with executive functions and self-monitoring. Neuroimaging studies indicate a shared reduction in grey matter volume and altered task-induced activations in the superior and middle temporal gyri, and the inferior frontal gyrus. Preliminary neurochemical studies indicate a shared glutamatergic dysfunction in language-related brain regions. Genome-wide association database indicates an overlap in genes involved in the risk for schizophrenia and language functions. Despite this overlap, further mechanistic studies are needed to explain how phenomenological divergence occurs at the level of clinical expression. Considering the potential utility of using speech analysis tools in clinical practice, we call for the development and testing of a “Language Network Modulation” treatment for schizophrenia especially targeting FTD and AVHs. Bridging different levels of evidence and conducting highly-controlled and reproducible experimental studies related to the language system will be critical to reaching this goal.

## Supplementary information

Supplementary Materials
