# Supplementary material for: Language abnormalities in schizophrenia: binding core symptoms through contemporary empirical evidence
Source: Schizophrenia (Heidelb). 2022 Nov 12;8(1):95. doi: 10.1038/s41537-022-00308-x (PMC9653408; doi:10.1038/s41537-022-00308-x)
Supplement: Supplementary file 1 — Supplementary Materials [file 41537_2022_308_MOESM1_ESM.docx]

**Supplementary Materials of**

*Language abnormalities in schizophrenia: binding core symptoms through contemporary empirical evidence*

[Supplementary Methods 2](#_Toc114481638)

[Human Connectome Project (HCP) 3T resting-state fMRI preprocessing 2](#_Toc114481639)

[Quantitative analysis of network overlap between FTD and AVHs 2](#_Toc114481640)

[Supplementary Tables 3](#_Toc114481641)

[SI Table 1 3](#_Toc114481642)

[SI Table 2 6](#_Toc114481643)

# Supplementary Methods

## Human Connectome Project (HCP) 3T resting-state fMRI preprocessing

HCP resting-state fMRI minimal processing pipeline includes correction for gradient distortion, head motion, B_0_ distortion, registration to the MNI space, intensity normalization, and removal non-brain voxel ^1^. The fMRI data were denoised using FIX (FMRIB's ICA-based X-noisifier) approach ^2^, bandpass filtered (0.01-0.1 Hz) and spatially smoothed using a 4mm FWHM kernel.

## Quantitative analysis of network overlap between FTD and AVHs

We used an approach to numerically quantify the degree of overlap among the networks that can be retrieved from loci consistently implicated in FTD and AVHs ^3–5^. To this end, we first retrieved the loci shown to have the highest anatomical likelihood of association with AVH. We used these loci as seed regions, and generated seed-specific networks from the Human Connectome Project (HCP) 3T resting-state fMRI data. Brain regions connected to the seed regions of each meta-analysis were determined by comparing *z*-transformed connectivity with zeros using one-sample *t*-test. T-statistic maps of each meta-analysis were set at a range of thresholds (T = 5 ~ 40, corresponding *p* = 1.7×10^-6^ ~ 1.8×10^-215^, *df* = 1095), and binarized to create a group mask (above 60% of studies). We calculated the Dice Similarity Coefficient to quantify overlap between brain network retrieved from each meta-analysis and the group mask. Dice index calculated as 2 times number of overlapping voxel and divided by total number of voxels in the two maps ^4^. We followed the 'overlap approach' from Peng and colleagues ^3^.

# Supplementary Tables

SI Table 1 Reported loci from meta-analyses of formal thought disorder and auditory verbal hallucinations*

| **Author** | **Year** | **X** | **Y** | **Z** | **Size** | **Unit** | **Coordinates** |
| --- | --- | --- | --- | --- | --- | --- | --- |
| Wensing et al. | 2017 | -46 | -50 | -2 | 71 | voxels | MNI |
|  |  | -54 | -28 | 4 | 110 | voxels | MNI |
|  |  | -56 | -56 | 12 | 109 | voxels | MNI |
| Modinos et al. | 2013 | -52 | -18 | 2 | 1680 | mm3 | MNI |
|  |  | -44 | -22 | 12 |  | mm3 | MNI |
|  |  | -46 | -14 | 6 |  | mm3 | MNI |
|  |  | 50 | -14 | 6 | 1248 | mm3 | MNI |
|  |  | 46 | -16 | -8 |  | mm3 | MNI |
|  |  | 50 | -14 | -10 |  | mm3 | MNI |
| Palaniyappan et al. | 2012 | -42 | -4 | 2 | 717 | voxels | Talairach |
|  |  | 58 | -6 | 10 | 318 | voxels | Talairach |
| Jardri et al. | 2011 | -48 | 10 | 7 | 1312 | mm3 | Talairach |
|  |  | -42 | 0 | 6 | 1240 | mm3 | Talairach |
|  |  | -54 | 0 | 14 | 488 | mm3 | Talairach |
|  |  | -24 | -32 | -4 | 1664 | mm3 | Talairach |
|  |  | 44 | 6 | -4 | 964 | mm3 | Talairach |
|  |  | 42 | 12 | -10 | 265 | mm3 | Talairach |
|  |  | -54 | -44 | 16 | 800 | mm3 | Talairach |
|  |  | -52 | -20 | 15 | 304 | mm3 | Talairach |
| Kompus et al. | 2011 | -44 | -2 | 6 | 2656 | mm3 | Talairach |
|  |  | -24 | -32 | -4 | 1064 | mm3 | Talairach |
|  |  | -50 | -24 | 40 | 1016 | mm3 | Talairach |
|  |  | 32 | -40 | 48 | 960 | mm3 | Talairach |
|  |  | -52 | -22 | 16 | 952 | mm3 | Talairach |
|  |  | 40 | 12 | 16 | 408 | mm3 | Talairach |
|  |  | 54 | -32 | -4 | 368 | mm3 | Talairach |
|  |  | 20 | -46 | -16 | 248 | mm3 | Talairach |
|  |  | 26 | 42 | 26 | 240 | mm3 | Talairach |
|  |  | 58 | -44 | 14 | 200 | mm3 | Talairach |
|  |  | -54 | -8 | 0 | 1824 | mm3 | Talairach |
|  |  | -10 | 0 | 40 | 520 | mm3 | Talairach |
|  |  | 12 | -22 | 18 | 520 | mm3 | Talairach |
|  |  | 24 | 50 | 14 | 456 | mm3 | Talairach |
|  |  | -12 | -38 | 10 | 392 | mm3 | Talairach |
| Kühn and Gallinat | 2012 | -55 | -19 | 16 | 344 | mm3 | Talairach |
|  |  | -49 | -17 | 41 | 256 | mm3 | Talairach |
|  |  | 36 | -32 | 50 | 216 | mm3 | Talairach |
|  |  | -48 | 2 | 6 | 208 | mm3 | Talairach |
|  |  | -56 | -30 | 0 | 424 | mm3 | Talairach |
|  |  | -10 | 3 | 56 | 376 | mm3 | Talairach |
|  |  | -4 | 26 | 31 | 160 | mm3 | Talairach |
|  |  | -44 | -22 | 0 | 152 | mm3 | Talairach |
|  |  | -42 | 2 | 18 | 152 | mm3 | Talairach |
|  |  | -9 | 4 | 37 | 112 | mm3 | Talairach |
| Zmigrod et al. | 2016 | −46 | −18 | 44 | 2104 | mm3 | MNI |
|  |  | −52 | −22 | 50 |  | mm3 | MNI |
|  |  | −40 | −16 | 54 |  | mm3 | MNI |
|  |  | 54 | 12 | −6 | 1608 | mm3 | MNI |
|  |  | 48 | 8 | −12 |  | mm3 | MNI |
|  |  | −56 | 4 | 12 | 1392 | mm3 | MNI |
|  |  | −44 | 0 | 4 |  | mm3 | MNI |
|  |  | −42 | 4 | −2 |  | mm3 | MNI |
|  |  | 60 | −22 | 44 | 1048 | mm3 | MNI |
|  |  | 64 | −16 | 36 |  | mm3 | MNI |
|  |  | 26 | −54 | −20 | 1024 | mm3 | MNI |
|  |  | 16 | −56 | −20 |  | mm3 | MNI |
|  |  | −12 | −20 | 4 | 888 | mm3 | MNI |
|  |  | −16 | −24 | −4 |  | mm3 | MNI |
|  |  | −26 | −32 | −4 | 792 | mm3 | MNI |
|  |  | −28 | −32 | 8 |  | mm3 | MNI |
|  |  | −58 | −46 | 20 | 792 | mm3 | MNI |
|  |  | −48 | −40 | 24 |  | mm3 | MNI |
|  |  | −60 | −56 | 20 |  | mm3 | MNI |
|  |  | 56 | 16 | 8 | 752 | mm3 | MNI |
|  |  | 50 | 24 | 0 |  | mm3 | MNI |
|  |  | 60 | 8 | 12 |  | mm3 | MNI |
|  |  | 6 | 6 | 60 | 664 | mm3 | MNI |
|  |  | −2 | 8 | 60 |  | mm3 | MNI |
|  |  | 26 | −62 | −46 | 560 | mm3 | MNI |
|  |  | 16 | −58 | −52 |  | mm3 | MNI |
|  |  | 40 | 0 | 12 | 352 | mm3 | MNI |
|  |  | 40 | −4 | 4 |  | mm3 | MNI |
|  |  | 0 | 12 | 46 | 344 | mm3 | MNI |
|  |  | 60 | −32 | −6 | 224 | mm3 | MNI |
|  |  | 18 | −10 | 2 | 216 | mm3 | MNI |

*****Meta-analyses were retrieved from PubMed search: *(“formal thought disorder” OR “auditory hallucinations” OR “auditory verbal hallucinations”) AND (neuroimaging OR (“brain imaging”) OR (“magnetic resonance imaging”) OR MRI OR (“positron emission tomography”) OR PET).* Article type was limited to meta-analyses published in English between January 1991 and August 2021.

SI Table 2 Overlap of brain networks from meta-analyses using dice similarity coefficient at different *t*-statistic thresholds

| Dice similarity coefficient***** | T>=5 | T>=10 | T>=20 | T>=30 | T>=40 |
| --- | --- | --- | --- | --- | --- |
| AVH_Jardri | 0.984 | 0.977 | 0.973 | 0.961 | 0.929 |
| AVH_Kompus_endogenous | 0.982 | 0.967 | 0.936 | 0.912 | 0.876 |
| AVH_Kompus_exogenous | 0.952 | 0.931 | 0.888 | 0.847 | 0.697 |
| AVH_Kuhn_State | 0.979 | 0.964 | 0.941 | 0.935 | 0.919 |
| AVH_Kuhn_Trait | 0.978 | 0.961 | 0.919 | 0.887 | 0.846 |
| AVH_Modinos_sMRI | 0.981 | 0.961 | 0.912 | 0.887 | 0.882 |
| AVH_Palaniyappan_sMRI | 0.981 | 0.970 | 0.956 | 0.953 | 0.938 |
| AVH_Zmigrod | 0.988 | 0.980 | 0.964 | 0.928 | 0.870 |
| FTD_Wensing | 0.975 | 0.955 | 0.908 | 0.882 | 0.780 |

*****Dice similarity coefficient is used to calculate overlap between brain network retrieved from each meta-analysis and a group mask generated from these networks. Dice index is calculated as number of overlapping voxel times 2, and divided by total number of voxels in the two maps.

**References**

1. Smith SM, Beckmann CF, Andersson J, et al. Resting-state fMRI in the Human Connectome Project. *Neuroimage*. 2013;80:144-168. doi:10.1016/j.neuroimage.2013.05.039

2. Salimi-Khorshidi G, Douaud G, Beckmann CF, Glasser MF, Griffanti L, Smith SM. Automatic denoising of functional MRI data: Combining independent component analysis and hierarchical fusion of classifiers. *Neuroimage*. 2014;90:449-468. doi:10.1016/j.neuroimage.2013.11.046

3. Peng S, Xu P, Jiang Y, Gong G. Activation network mapping for integration of heterogeneous fMRI findings. *Nat Hum Behav*. June 2022. doi:10.1038/s41562-022-01371-1

4. Darby RR, Joutsa J, Fox MD. Network localization of heterogeneous neuroimaging findings. *Brain*. 2019;142(1):70-79. doi:10.1093/brain/awy292

5. Boes AD, Prasad S, Liu H, et al. Network localization of neurological symptoms from focal brain lesions. *Brain*. 2015;138(10):3061-3075. doi:10.1093/brain/awv228
